# Strengthening the success rate of suprapubic aspiration in infants by integrating point-of-care ultrasonography guidance: A parallel-randomized clinical trial

**DOI:** 10.1371/journal.pone.0254703

**Published:** 2021-07-15

**Authors:** Sadroddin Mahdipour, Seyedeh Nastaran Seyed Saadat, Hamidreza Badeli, Afagh Hassanzadeh Rad

**Affiliations:** 1 Neonatologist, Pediatric Diseases Research Center, Guilan University of Medical Sciences, Rasht, Iran; 2 Pediatrician, Pediatric Diseases Research Center, Guilan University of Medical Sciences, Rasht, Iran; 3 Pediatric Nephrologist, Pediatric Diseases Research Center, Guilan University of Medical Sciences, Rasht, Iran; 4 BS of Midwifery, PhD of Linguistics, Pediatric Diseases Research Center, Guilan University of Medical Sciences, Rasht, Iran; The Chinese University of Hong Kong, HONG KONG

## Abstract

**Background:**

Urinary tract infection (UTI) is a common disease in childhood. A sterile collection of urine samples using suprapubic aspiration (SPA) and bladder catheterization (BC) is helpful for rapid and accurate diagnosis of UTI in infants. With the advent of point-of-care ultrasound (POCUS), the use of ultrasound by non-radiologists at the patient’s bedside, great advancement has been noticed in various medical fields. Considering the importance and advantages of using POCUS in the physical examination and guiding procedures, the authors aimed to compare urine sampling’s success rate by SPA, BC, and POCUS guided SPA (POCUS-SPA) in infants performed by three pediatricians.

**Materials and methods:**

This study is a randomized clinical trial conducted on 114 neonates and infants with suspected UTI admitted to 17-Shahrivar children’s hospital from April 2017 to September 2019. Neonates and infants were randomly assigned to three groups of BC, SPA, and POCUS-SPA. The primary outcome was the success of sampling defined by obtaining 1cc of urine in each method. The secondary outcome was assessing the pain level.

**Results:**

Results showed that the POCUS-SPA had the highest success rate in urine sampling, and a statistically significant relation was noted among the three groups (P = 0.0001). From 38 patients in each group, 37 patients of POCUS-SPA (97.4%), 34 patients of BC (89.5%), and 23 patients of SPA (60.5%) had a successful sampling. Most of the patients in all three groups experienced severe pain.

**Conclusions:**

In the current study, results showed that the POCUS-SPA significantly increased the success rate of urine sampling and most of the patients in all three groups had severe pain. Based on the shortage of access to radiologists in emergency setups, it seems that the POCUS-SPA by the pediatricians can be one of the most appropriate and applicable diagnostic methods in infants with UTI.

## Introduction

Urinary tract infection (UTI) is a common disease in childhood and may cause complications such as pyelonephritis, bacteremia, urosepsis, renal scar, hypertension, and end-stage renal disease. Early diagnosis and antibiotic prescription can considerably prevent UTI-related severe complications [1,2]. Urine culture is the primary gold standard for the diagnosis of UTI.

Urine sampling methods in infants who are not toilet trained include urine collection pads, clean catch, clean urine bag specimen, bladder catheterization (BC), and suprapubic aspiration (SPA). Urinary contamination of collected urine is one of the clinician’s most important concerns. Among different urine collection methods, the contamination rates of SPA, BC, and clean catch are respectively 1% [3], 6–12% [3–5], and 16–63% [3–6].

In contrast to urine collection pads, clean catch, and clean urine bag specimens, BC and SPA are invasive methods with low contamination rates. Invasive techniques are indicated if a urine sample can not be obtained using non-invasive methods in critically ill infants [7,8]. Based on the many international UTI guidelines, in well children, a urine bag is the first choice of sampling [9–12]. Besides, in ill children, BC and SPA are the methods of choice [7–9]. Alternatives to invasive collection methods are voiding stimulation for a clean catch like the Quick-Wee [13] and bladder-lumbar stimulation [14] methods, which have a relatively admissible success rate.

Estimation of the quantity of urine in the bladder can increase the success rate of urine sampling in SPA. Therefore, ultrasound (US) can be used to measure the quantity of urine in the bladder. A previous study indicated that integrating real-time ultrasound (RTUS) for detecting urine sufficiency by SPA could increase sampling success rate. Their results showed that RTUS compared to automated bladder ultrasound (ABUS) had superior repeatability and higher accuracy in bladder volume measurements in children aged < 24 months. Therefore, they mentioned that ABUS was not a reliable method before bladder instrumentation [15]. Despite the applicability of RTUS, the shortage of access to radiologists in emergency setups should be considered.

Regarding this shortage, point-of-care ultrasound (POCUS) by physicians is used in many medical specialties and subspecialties [16]. Considering the importance of using POCUS at the patients’ bedside in the physical examination and guiding procedures, there is a lack of research comparing the success rate of SPA, BC, and POCUS guided SPA (POCUS-SPA) by the pediatricians who treat newborns and infants in the emergency departments. Therefore, the authors aimed to compare urine sampling’s success rate by SPA, BC, and POCUS-SPA in infants performed by three pediatricians. In case of successful sampling, POCUS-SPA can help pediatricians to make rapid and proper decisions and early treatment.

## Materials and methods

This study is a non-blinded-paralleled-randomized clinical trial conducted on 114 neonates and infants with suspected UTI admitted to 17-Shahrivar children’s hospital from April 2017 to September 2019. Neonates and infants younger than three months were eligible to include in this study. Suspicious of UTI was determined by fever without source, a workup for sepsis, and hyperbilirubinemia. Exclusion criteria were cutaneous infection at the suprapubic area, enlarged intra-abdominal viscera, known anatomical disorders of the urinary tract, bleeding disorders, uncorrected thrombocytopenia, and enlarged abdomen.

Patients were randomly divided into three groups A, B, and C. Group A underwent BC by a pediatrician. Group B underwent SPA by a neonatologist. Group C underwent POCUS-SPA by an experienced pediatric nephrologist in performing POCUS. All patients were fed with oral sucrose) 2 cc(before the procedure for pain reduction. To reduce the investigator bias of sampling by a single clinician in all three groups, three different clinicians who had more than five years’ experience in performing each procedure were included. In each group, one nurse was engaged to aid the clinician throughout the procedure. There is no extra charge for performing POCUS in the setting of Iranian governmental hospitals.

For POCUS-SPA, a linear US probe (Ultrosonix machine, Canada) was used to assess the presence of adequate urine and visualize *the needle’s tip in the path of real-time aspiration*. In the SPA and POCUS-SPA groups, 10 cc syringe with a 24-gauge needle was used, and 30 minutes before the aspiration, the region was locally anesthetized by Lidocaine cream. In case of no urine aspiration in the SPA group, or unsuccessful sampling in the SPA and POCUS-SPA groups, the procedure was repeated 30 minutes after the first attempt.

The collected data included age (days), sex, weight (gram), length (centimeter), the number of urinary intervention attempts, duration of sampling (second), the complications of each method, contamination rate (the existence of ≥ 2 different organisms in the urine culture), pain level, and duration of crying (seconds). The primary outcome was considered as the success of sampling that was defined by obtaining 1cc of urine in each method. Unsuccessful sampling was indicated if there was no successful sampling within three consecutive attempts for each patient. Secondary outcomes were duration of sampling, pain score, and duration of crying.

The duration of sampling was calculated from introducing the instruments (needle or catheter, and probe contact with the skin) until the completion of urine sample collection. In the case of several attempts, the cumulative duration was reported.

Besides, the neonatal infant pain scale (NIPS), a reliable and valid method for pain assessment [17,18], was used to determine each patient’s pain score. NIPS assessed items including facial expression, crying, breathing patterns, arms and legs movement, and state of arousal and estimated the pain level between 0 to 7 (0–2 = mild to no pain, 3–4 = mild to moderate pain, >4 = severe pain) (Table 1).

**Table 1:** The neonatal infant pain scale.

Factors	Findings	Score
Facial expression	Relaxed	0
	Grimace	1
Cry	No cry	0
	Whimper	1
	Vigorous	2
Breathing pattern	Relaxed	0
	Change in breathing	1
Extremity movement	Relaxed/restrained	0
	Flexed/extended	1
State of arousal	Sleeping/awake	0
	Fussy	1

As there is no previous study that compared the success rate of using POCUS-SPA with other routine methods, based on the semi-similar previous research by Badiee et al. [19], the sample size for each group was calculated. They compared the success rate of SPA without POCUS and BC in premature uncircumscribed boys, and it was indicated that 38 patients should be included in each group.

1-∝ = 95%

Z(1- ∝/2) = 1.96

Β = 20%

Z 1-β = 0.84

P1 = 71% (success rate of SPA) P2 = 53% (success rate of BC)

d = 0.3

$n=\frac{{\left(Z_{1-\frac{\alpha }{2}}+Z_{1-\beta }\right)}^{2}[(p1(p1-p2)+p2(1-p2)]}{d^{2}}n=\frac{{\left(Z_{1-\frac{\alpha }{2}}+Z_{1-\beta }\right)}^{2}[(p1(p1-p2)+p2(1-p2)]}{d^{2}}$

$n=\frac{{\left(1.96+0.84\right)}^{2}[0.71(1-0.71)+0.53(1-0.53)]}{0.3^{2}}n=\frac{{\left(1.96+0.84\right)}^{2}[0.71(1-0.71)+0.53(1-0.53)]}{0.3^{2}}$

n = 38 per group

Randomization was performed by the randomization blocking method. An unaware nurse assigned participants to three groups. In each group, patients were visited 6–8 and 48 hours after performing the procedure by the clinician for assessing the complications. The main complications of BC were indicated as macroscopic hematuria, urinary retention, and urethral bleeding. Subdermal hematoma, contamination of the sample with more than two bacterial germs on culture, and intestinal perforation within 6 hours or evidence of air in abdominal x-ray were indicated as SPA’s probable complications.

### Ethical considerations

Written informed consent was taken from parents or guardians. This study was approved by the ethics committee of Guilan University of Medical Sciences (number: IR.GUMS.REC.1395.365 date: 2016-12-15), and an RCT code was obtained (IRCT20090111001545N3).

### Statistical analysis

The data were reported by descriptive statistics such as number, percent, mean, and standard deviation. The normality of quantitative data was assessed by the Kolmogorov-Smirnov test. Based on the non-normal distribution of quantitative variables, the Kruskal Wallis test was used. Qualitative variables were analyzed by chi-square and Fisher’s exact tests in the SPSS version 19. P-value< 0.05 indicated the statistical significance.

## Results

The results showed that 68 (59.60%) boys and 46 (40.4%) girls with the mean age of 35.68±14.23 days participated in this study. The mean weight and length were 4198.2±1259 grams and 52.95±84.3 centimeters, respectively. There was no statistically significant relationship among the three groups regarding age, sex, weight, and height (p>0.05).

**Fig 1:**
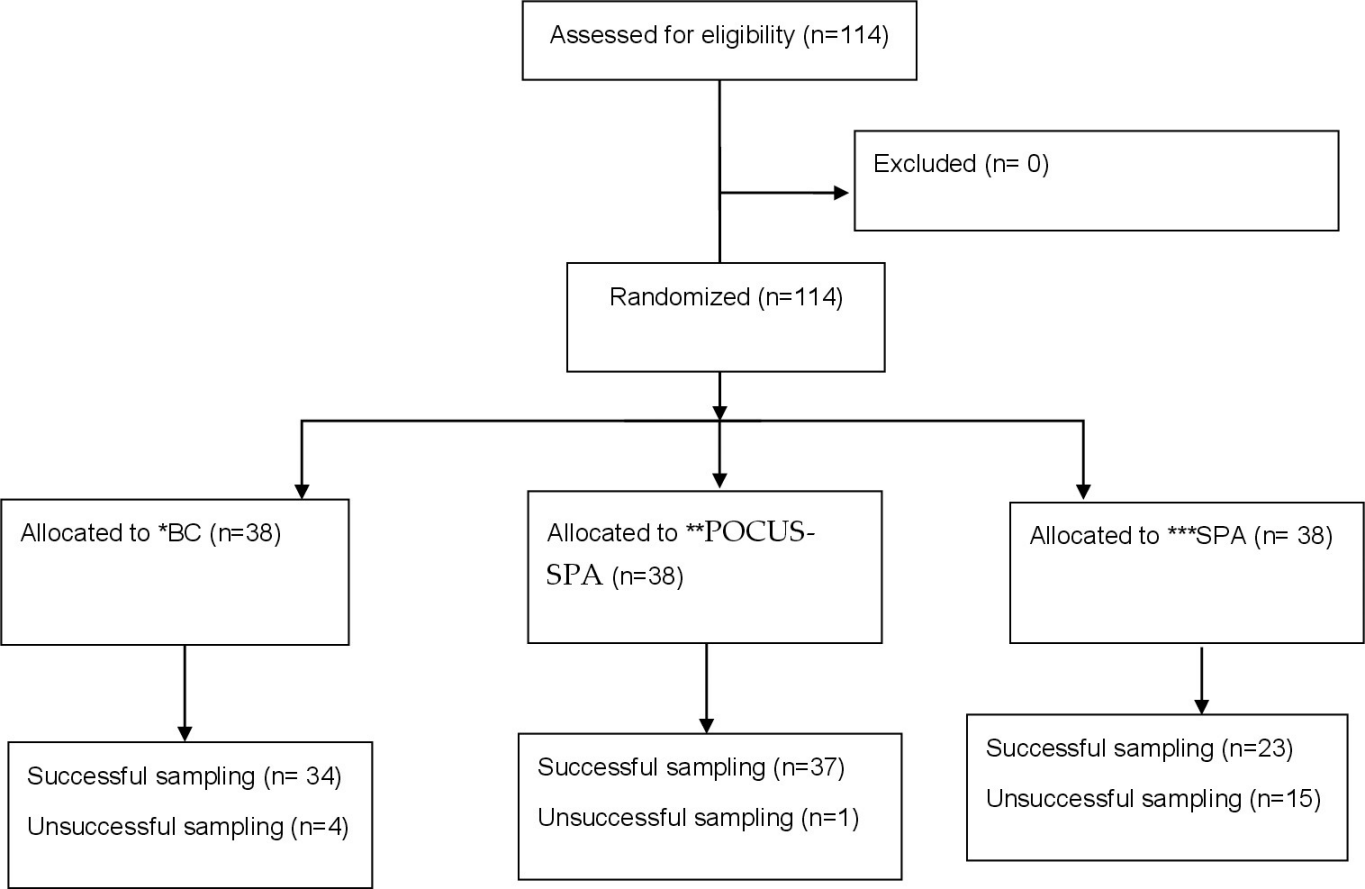
The CONSORT flow diagram.

Considering 1 cc urine sample as the successful performance, results showed that POCUS-SPA had the highest success rate in urine sampling among the three groups (P = 0.0001). From 38 patients in each group, 37 patients of POCUS-SPA (97.4%), 34 patients of BC (89.5%), and 23 patients of SPA (60.5%) groups had successful sampling (Fig 1).

In POCUS-SPA, SPA, and BC groups, respectively, 35 out of 37 (94.6%), 16 out of 23 (69.6%), and 19 out of 34 successful cases (55.9%) had successful sampling within the first attempt. ’ ’Fisher’s exact test demonstrated a statistically significant relationship due to the number of attempts in three methods. It showed that most POCUS-SPA’s successful sampling was significantly achieved in the first attempt (P = 0.0001).

In terms of duration of sampling, the highest duration belonged to BC (104.9±43.1 seconds). Moreover, the duration of sampling in POCUS-SPA was 23.61 ± 11.8 seconds and in SPA was 15.89±5.23 seconds. Using the Kruskal Wallis test showed a statistically significant difference (P = 0.0001).

In terms of the crying duration, BC had the longest (45.39±32.117 seconds), and SPA had the shortest duration (18.62±2.6 seconds). Besides, the duration of crying by the POCUS-SPA method was 34.97± 20.45 seconds. Kruskal Wallis test showed a statistically significant difference in terms of duration of crying among the three methods (P = 0.0001).

In the case of assessing contamination rate, results showed no contaminated urinary samples in all three groups.

NIPS showed that most of the patients in all three groups experienced severe pain. Severe pain was noted respectively in 78.9% of BC, 63.2% of POCUS-SPA, and 55.3% of SPA. Using the Chi-square test determined no statistically significant relationship between the severity of pain among groups (p = 0.183) (Table 2).

**Table 2:** The number of successful sampling attempts and the severity of pain using the three methods.

	BC Num(%)	POCUS-SPA Num(%)	SPA Num(%)	p-value
**Number of attempts for SS**				P = 0.001
1 attempt	19(55.9)	35(94.6)	16(69.6)	
2 attempts	12(35.3)	2(5.4)	7(30.4)	
3 attempts	3(8.8)	0	0	
total	34 (100)	37 (100)	23 (100)	
**The severity of pain**				P = 0.183
No pain(0–2)	6(15.8)	7(18.4)	11(28.9)	
Moderate pain(3–4)	2(5.3)	7(18.4)	6(15.8)	
Severe pain(>4)	30(78.9)	24(63.2)	21(55.3)	
total	38(100)	38(100)	38(100)	

The highest pain score was noted by BC (5.23 ±1.95). The pain score in the POCUS-SPA group was 4.78±1.91 and in the SPA was 4.13±1.98. Kruskal Wallis test showed a statistically significant difference in obtained scores by three methods (P = 0.0027). Results showed no complication during follow-up in groups.

## Discussion

In this study, the success rate of three different methods of infant urine sampling, including BC, SPA, and POCUS-SPA, were compared. Results indicated that performing POCUS-SPA by the physician had the highest success rate (97.4%) than the other two methods (89.5% for BC and 60.5% for SPA). Although SPA had the least pain score among three methods, results showed that most of the patients in all three groups had severe pain, therefore the highest success rate of POCUS-SPA was remarkable and it seems that POCUS-SPA can be one of the most appropriate diagnostic methods in infants with UTI.

Considering the lack of research comparing SPA, BC, and POCUS-SPA’s success rate by a pediatrician in the emergency department, the authors compared the current results with previous semi-similar studies. Badiee et al., who compared the success rate of SPA without POCUS and BC in only premature uncircumscribed boys, reported that the success rate of urine sampling by SPA was 53% compared to 71% for BC [19]. In other studies comparing BC and SPA by Pollack et al. on infants younger than six months and Austin et al. on neonates, BC had a higher success rate than SPA (100% vs.46%, and 77% vs.67%, respectively) [20,21].

In addition to the previous studies that compared the success rate of sampling by SPA and BC, radiologists attempted to strengthen the success rate by adding US for determining the presence of urine in the bladder before sampling. O’Callaghan and McDougall reported a significantly higher success rate for US-guided SPA performed by radiologists (100%) compared to SPA alone (36%) [22]. Similarly, Gochman et al. reported 79% success in US-guided SPA compared to 52% of SPA [23]. Ozkan et al. also mentioned consistent results. They demonstrated that US-guided SPA had a higher success rate than SPA alone (90% vs.64%) [24]. Despite the higher success rate of previous research performed by radiologists, based on the lack of 24-hour access to radiologists, the current study mainly aimed to evaluate the significance of POCUS, performing US by a clinician at the bedside.

The alternative method for successful sampling is ABUS. It is a simple, quick, easily applicable, and accurate device for bladder volume content measurement. A study by Munir et al., which measured the bladder volume by ABUS, showed that from 37 subjects that underwent ABUS before SPA, 90% had successful SPA [25]. Bevan et al. showed that RTUS compared to ABUS had superior repeatability and higher accuracy in bladder volume measurements in children aged < 24 months. Therefore, they concluded that ABUS was not a reliable method before bladder instrumentation [15]. Add to their conclusions, ABUS, a device that only measures bladder volume with no visualization of the needle’s tip in the path of real-time aspiration, imposes extra expenses in comparison with US as a multifunctional device.

Moreover, clinicians can apply non-invasive collection methods such as bladder-lumbar stimulation [14]. Based on the previous study, this method had a high success rate in neonates aged < 7 days old (90%) [26], however, its success rate decreased with increasing age. Results showed that in those aged < 6 months, the success rate was estimated as 49% [4]. The Quick-Wee technique is another non-invasive method of voiding stimulation with 31% success rate [13]. Despite the non-invasiveness of these methods, they are not suitable for urine collection in the critical situation regarding their low success rate.

In this study, the pain was another item that was assessed. Most of the patients experienced severe pain based on NIPS in all three methods. The highest pain score was noted by the BC, followed by the POCUS-SPA and the SPA. Furthermore, the longest and shortest duration of crying were reported by the BC and the SPA, respectively. Similar to this study, Badiee et al. mentioned that a higher score of pain existed in the BC method than SPA. However, in the case of crying duration, they noted that the average time of crying was longer in the SPA group than the BC group (77 vs. 34.4 seconds) [19]. Kozer et al. reported a shorter duration of crying in the BC group compared to SPA with no significant correlation between methods [27]. This difference may be occurred due to the complexity of performing BC and POCUS-SPA in comparison with SPA. POCUS-SPA needs steps such as fixation, detecting urine in the bladder by US, sterile prep and draping, and finally sampling under the guidance of US. In addition, BC needs sterile prep and draping, catheterization, and waiting for sufficient urine collection. In contrast, SPA only needs fixation, sterile prep and draping, and sampling. The authors assumed that a longer duration of the process (from fixation to visualization and sampling) caused higher pain scores and a longer duration of crying. This study had some limitations. The authors did not detect urine volume by US before BC and SPA. In addition, the authors assessed the duration of the total procedure and did not separately measure the duration of the sampling step, immediately after introducing the needle or catheter.

## Conclusions

In the current study, results showed that POCUS-SPA significantly increased the success rate of urine sampling. Although SPA had the least pain score among three methods, most of the patients in all three groups had severe pain, therefore the highest success rate of performing POCUS-SPA by the pediatrician was remarkable. Based on the shortage of access to radiologists in emergency setups, it seems that POCUS-SPA can be one of the most appropriate diagnostic methods that lead to rapid decision-making and early treatment in infants with UTI.

## Supporting information

S1 Checklist CONSORT 2010 checklist of information to include when reporting a randomised trial*. (DOC)

S1 File (PDF)

S2 File (PDF)

S3 File (DOCX)
