# Supplementary material for: Strengthening the success rate of suprapubic aspiration in infants by integrating point-of-care ultrasonography guidance: A parallel-randomized clinical trial
Source: PLoS One. 2021 Jul 15;16(7):e0254703. doi: 10.1371/journal.pone.0254703 (PMC8282064; doi:10.1371/journal.pone.0254703)
Supplement: S1 File — (PDF) [file pone.0254703.s002.pdf]

Clinical Trial Protocol
Iranian Registry of Clinical Trials

05 Dec 2020

Comparing the success rate of the three urine sampling methods with Catheterization, Suprapubic Aspiration with and without Guidance in Neonates and Infants younger than three month suspicious to Urinary Tract Infection

Protocol summary

Study aim
To determine and compare the success rate of three urine sampling methods with catheterization, suprapubic aspiration with and without guidance in infants suspicious to urinary tract infection
Design
clinical trial with control group
Settings and conduct
Neonates and infants hospitalized in 17 Shahrivar Hospital suspicious to Urinary Tract Infection are divided into 3 therapeutic groups: A, B, and C according to block randomization. Group A: Sampling with Catheterization Group B: Sampling with Suprapubic Aspiration Group C: Sampling with Ultrasonography-guided Suprapubic Aspiration There is a maximum limit of three-time efforts for sampling
Participants/Inclusion and exclusion criteria
Entry condition: All the Neonates and Infants younger than 3 month suspicious to Urinary Tract Infection No-entry conditions: Infection of the examed -skin site Anatomical disorders of Urinary Tract Bleeding Disorders and Uncorrected Thrombocytopenia Enlarged abdomen and Inrta-abdominal Viscera
Intervention groups
Group A: Catheterization Group B: Suprapubic Aspiration Group C: Ultrasonography-guided Suprapubic Aspiration
Main outcome variables
Overall success rate of each sampling method

General information

Reason for update
Acronym
IRCT registration information
IRCT registration number: **IRCT20090111001545N3**
Registration date: **2018-04-09, 1397/01/20**
Registration timing: **registered_while_recruiting**

Last update: **2018-04-09, 1397/01/20**
Update count: **0**
Registration date
2018-04-09, 1397/01/20

Registrant information
Name
Hamidreza Badeli
Name of organization / entity
Guilan University of Medical Sciences
Country
Iran (Islamic Republic of)
Phone
+98 13 1322 9523
Email address
badeli@gums.ac.ir

Recruitment status
Recruitment complete
Funding source

Expected recruitment start date
2017-12-22, 1396/10/01
Expected recruitment end date
2018-12-22, 1397/10/01
Actual recruitment start date
empty
Actual recruitment end date
empty
Trial completion date
empty

Scientific title
Comparing the success rate of the three urine sampling methods with Catheterization, Suprapubic Aspiration with and without Guidance in Neonates and Infants younger

than three month suspicious to Urinary Tract Infection

Public title
Comparing the success rate of the three urine sampling methods with Catheterization, Suprapubic Aspiration with and without Guidance in neonates and infants younger than three month with Urinary Tract Infection

Purpose
Diagnostic

Inclusion/Exclusion criteria

Inclusion criteria:
All the Neonates and Infants younger than 3 month suspicious to Urinary Tract Infection

Exclusion criteria:
Infection of the examed -skin site Anatomical disorders of Urinary Tract Bleeding Disorders and Uncorrected Thrombocytopenia Enlarged abdomen and Intra-abdominal Viscera

Age
From **1 day** old to **3 months** old

Gender
Both

Phase
2-3

Groups that have been masked
No information

Sample size
Target sample size: **114**

Randomization (investigator's opinion)
Randomized

Randomization description
Sampling will be performed by randomization method. Neonates and infants will be classified into 3 therapeutic groups by block randomization.

Blinding (investigator's opinion)
Not blinded

Blinding description

Placebo
Not used

Assignment
Parallel

Other design features

Secondary Ids

empty

Ethics committees

1

Ethics committee

Name of ethics committee
Ethics committee of Guilan University of Medical Sciences

Street address
Namjou Ave,Shadid Siadati St,Rasht

City
Rasht

Province
Guilan

Postal code
4144654839

Approval date
2016-12-15, 1395/09/25

Ethics committee reference number
IR.GUMS.REC.1395.365

Health conditions studied

1

Description of health condition studied
Success rate of urine sampling methods

ICD-10 code
P39.3

ICD-10 code description
Neonatal urinary tract infection

Primary outcomes

1

Description
Overall success rate of each sampling method

Timepoint
Three times

Method of measurement
Catheterization,Suprapubic aspiration without guidance,Suprapubic aspiration with guidance

Secondary outcomes

empty

Intervention groups

1

Description
Intervention group 1: The group in which urine sample is collected by Catheterization

Category
Diagnosis

2

Description
Intervention group 2: The group in which urine sample is collected by Suprapubic Aspiration

Category
Diagnosis

3

Description
Intervention group 3: The group in which urine sample is collected by Ultrasonography-guided suprapubic Aspiration

Category
Diagnosis

Recruitment centers

1

Recruitment center
Name of recruitment center
17-Shahrivar Children Hospital
Full name of responsible person
Hamidreza Badeli
Street address
Namjou Ave,Shadid Siadati St,Rasht
City
Rasht
Province
Guilan
Postal code
41144654839
Phone
+98 13 3336 9002
Fax
+98 13 3336 9070
Email
17shahrivar@gums.ac.ir
Web page address

Sponsors / Funding sources

1

Sponsor
Name of organization / entity
Rasht University of Medical Sciences
Full name of responsible person
Shadman Nemati
Street address
Namjou Ave, Shadid Siadati St, Rasht
City
Rasht
Province
Guilan
Postal code
4144654839
Phone
+98 13 3336 9002
Fax
Email
research@gums.ac.ir
Web page address
Grant name
Grant code / Reference number
Is the source of funding the same sponsor organization/entity?
Yes
Title of funding source
Rasht University of Medical Sciences
Proportion provided by this source
100
Public or private sector
Public
Domestic or foreign origin
Domestic
Category of foreign source of funding
empty
Country of origin
Type of organization providing the funding
Academic

Person responsible for general inquiries

Contact
Name of organization / entity
Rasht University of Medical Sciences
Full name of responsible person
Hamidreza Badeli
Position
Assistant professor
Latest degree
Subspecialist
Other areas of specialty/work
Pediatrics
Street address
Namjou Ave,Shadid Siadati St,Rasht
City
Rasht
Province
Guilan
Postal code
4144654839
Phone
+98 13 3336 9002
Fax
+98 13 3336 9070
Email
badeli@gums.ac.ir

Person responsible for scientific inquiries

Contact
Name of organization / entity
Rasht University of Medical Sciences
Full name of responsible person
Hamidreza Badeli
Position
Assistant Professor
Latest degree
Subspecialist
Other areas of specialty/work
Pediatrics
Street address
Namjou Ave,Shadid Siadati St,Rasht
City
Rasht
Province
Guilan
Postal code
4144654839
Phone
+98 13 3336 9391
Fax
Email
badeli@gums.ac.ir

Person responsible for updating data

Contact

Name of organization / entity
Rasht University of Medical Sciences
Full name of responsible person
Hamidreza Badeli
Position
Associate Professor
Latest degree
Subspecialist
Other areas of specialty/work
Pediatrics
Street address
Namjou Ave,Shadid Siadati St,Rasht
City
Rasht
Province
Guilan
Postal code
414465839
Phone
+98 13 3336 9002
Email
badeli@gums.ac.ir

Sharing plan

Deidentified Individual Participant Data Set (IPD)
No - There is not a plan to make this available
Justification/reason for indecision/not sharing IPD
regarding the ethical issues and confidentiality
Study Protocol
No - There is not a plan to make this available
Statistical Analysis Plan
No - There is not a plan to make this available
Informed Consent Form
No - There is not a plan to make this available
Clinical Study Report
No - There is not a plan to make this available
Analytic Code
No - There is not a plan to make this available
Data Dictionary
No - There is not a plan to make this available
