# Supplementary material for: Strengthening the success rate of suprapubic aspiration in infants by integrating point-of-care ultrasonography guidance: A parallel-randomized clinical trial
Source: PLoS One. 2021 Jul 15;16(7):e0254703. doi: 10.1371/journal.pone.0254703 (PMC8282064; doi:10.1371/journal.pone.0254703)
Supplement: S2 File — (PDF) [file pone.0254703.s003.pdf]

پروتکل کارآزمایی بالینی
مرکز ثبت کارآزمایی بالینی ایران

۱۳۹۹/۰۹/۱۵

مقایسه میزان موفقیت سه روش نمونه گیری ادرار با کاتتر آسپیراسیون سوپراپوبیک با و بدون گایدنس در نوزادان و شیرخواران کمتر از سه ماه مشکوک به عفونت ادراری

چکیده پروتکل

هدف ازمطالعه

تعیین و مقایسه میزان موفقیت سه روش نمونه گیری ادرار با کاتتر آسپیراسیون سوپراپوبیک با و بدون گایدنس در شیرخواران مشکوک به عفونت ادراری

طراحی

کارآزمایی بالینی دارای گروه کنترل

نحوه و محل انجام مطالعه

نوزادان و شیرخواران بستری شده در بیمارستان 17 شهریور مشکوک به عفونت ادراری 30 دقیقه پس از شیرخوردن بر اساس بلاک های تصادفی به سه گروه درمانی A,B ,C تقسیم بندی میشوند. گروه A : نمونه گیری با کاتترمثانه گروه B:نمونه گیری با آسپیراسیون سوپراپوبیک گروه C:نمونه گیری سوپراپوبیک با گاید اولتراسونوگرافی تعداد دفعات تلاش برای نمونه گیری حداکثر سه بار است

شرکت کنندگان/شرایط ورود و عدم ورود

شرایط ورود: کلیه نوزادان و شیرخواران کوچکتر از سه ماه مشکوک به عفونت ادراری شرایط عدم ورود: عفونت پوست محل مورد بررسی اختلالات آناتومیک دستگاه ادراری اختلالات خونریزی دهنده و ترومبوسیتوپنی اصلاح نشده شکم و احشا شکمی برجسته

گروه‌های مداخله

گروه A : نمونه گیری با کاتترمثانه گروه B:نمونه گیری با آسپیراسیون سوپراپوبیک گروه C:نمونه گیری سوپراپوبیک با گاید اولتراسونوگرافی

متغیرهای پیامد اصلی

میزان موفقیت کلی هر روش نمونه گیری ادرار

اطلاعات عمومی

علت بروز رسانی

نام اختصاری

اطلاعات ثبت در مرکز

شماره ثبت کارآزمایی در مرکز: **IRCT20090111001545N3**

تاریخ تایید ثبت در مرکز: **2018-04-09, ۱۳۹۷/۰۱/۲۰**

زمان‌بندی ثبت: **registered_while_recruiting**

آخرین بروز رسانی: **2018-04-09, ۱۳۹۷/۰۱/۲۰**

تعداد بروز رسانی‌ها: **0**

تاریخ تایید ثبت در مرکز

2018-04-09, ۱۳۹۷/۰۱/۲۰

اطلاعات تماس ثبت کننده

نام

حمیدرضا بادلی

نام سازمان / نهاد

دانشگاه علوم پزشکی گیلان ، مرکز آموزشی درمانی 17 شهریور رشت

کشور

جمهوری اسلامی ایران

تلفن

+98 13 1322 9523

آدرس ایمیل

badeli@gums.ac.ir

وضعیت بیمار گیری

بیمار گیری تمام شده

منبع مالی

تاریخ شروع بیمار گیری مورد انتظار

2017-12-22, ۱۳۹۶/۱۰/۰۱

تاریخ پایان بیمار گیری مورد انتظار

2018-12-22, ۱۳۹۷/۱۰/۰۱

تاریخ شروع بیمارگیری تحقق یافته

خالی

تاریخ پایان بیمارگیری تحقق یافته

خالی

تاریخ خاتمه کارآزمایی

خالی

عنوان علمی کارآزمایی

مقایسه میزان موفقیت سه روش نمونه گیری ادرار با کاتتر آسپیراسیون سوپراپوبیک با و بدون گایدنس در نوزادان و شیرخواران کمتر از سه ماه مشکوک به عفونت ادراری

عنوان عمومی کارآزمایی

مقایسه میزان موفقیت سه روش نمونه گیری ادرار با کاتتر آسپیراسیون سوپراپوبیک با و بدون گایدنس در نوزادان و شیرخواران کمتر از سه ماه مشکوک به عفونت ادراری

هدف اصلی مطالعه

تشخیصی

شرایط عمده ورود و عدم ورود به مطالعه

شرایط عمده ورود به مطالعه قبل از تصادفی سازی:

کلیه نوزادان و شیرخواران کوچکتر از سه ماه مشکوک به عفونت

ادراری
شرایط عمده عدم ورود به مطالعه قبل از تصادفی سازی:
عفونت پوست محل مورد بررسی اختلالات آناتومیک دستگاه ادراری اختلالات خونریزی دهنده و ترومبوسیتوپنی اصلاح نشده شکم و احشا شکمی برجسته

سن
از سن **1 روزه** تا سن **3 ماهه**

جنسیت
هر دو

فاز مطالعه
2-3

گروه‌های کور شده در مطالعه
اطلاعات موجود نیست

حجم نمونه کل
حجم نمونه پیش‌بینی شده: **114**

تصادفی سازی (نظر محقق)
اختصاص تصادفی به گروه‌های مداخله و کنترل

توصیف نحوه تصادفی سازی
نمونه گیری به روش تصادفی خواهد بود، نوزادان و شیرخواران بر اساس block های تصادفی به سه گروه درمانی تقسیم بندی می شوند

کور سازی (به نظر محقق)
کور نشده است

توصیف نحوه کور سازی

دارو نما
ندارد

اختصاص به گروه‌های مطالعه
موازی

سایر مشخصات طراحی مطالعه

کد ثبت در سایر مراکز ثبت بین‌المللی

خالی

تاییدیه کمیته‌های اخلاق

1

کمیته اخلاق

نام کمیته اخلاق
کمیته اخلاق دانشگاه علوم پزشکی گیلانٍ

آدرس خیابان
بیمارستان 17 شهریور.خیابان شهید سیادتی.خیابان نامجو

شهر
رشت

استان
گیلان

کد پستی
4144654839

تاریخ تایید
2016-12-15, ۱۳۹۵/۰۹/۲۵

کد کمیته اخلاق
IR.GUMS.REC.1395.365

بیماری‌های (موضوعات) مورد مطالعه

1

شرح
میزان موفقیت روش های نمونه گیری ادرار

کد ICD-10
P39.3

توصیف کد ICD-10
Neonatal urinary tract infection

متغیر پیامد اولیه

1

شرح متغیر پیامد
میزان موفقیت کلی هر روش نمونه گیری ادرار

مقاطع زمانی اندازه‌گیری
سه بار

نحوه اندازه‌گیری متغیر
کاتترگذاری گذاری-آسّپیراسیون سوپراپوبیک بدون گاید سونوگرافی-آسپیران سوپراپوبیک با گاید سونوگرافی

متغیر پیامد ثانویه

خالی

گروه‌های مداخله

1

شرح مداخله
گروه مداخله اول: گروهی که نمونه ادرار توسط کاتترگذاری مثانه جمع آوری میشود

طبقه بندی
تشخیصی

2

شرح مداخله
گروه مداخله دوم: گروهی که نمونه ادرار توسط آسپیراسیون سوپراپوبیک جمع آوری میشود

طبقه بندی
تشخیصی

3

شرح مداخله
گروه مداخله سوم: گروهی که نمونه ادرار توسط آسپیراسیون سوپراپوبیک همراه گایدسونوگرافی جمع آوری میگردد

طبقه بندی
تشخیصی

مراکز بیمار گیری

1

مرکز بیمار گیری

نام مرکز بیمار گیری
بیمارستان کودکان 17-شهریور

نام کامل فرد مسوول
حمیدرضا بادلی

آدرس خیابان
بیمارستان 17 شهریور-خیابان نامجو-خیابان شهید سیادتی-روبروی پارک شهر

شهر
رشت

استان
گیلان

کد پستی

41144654839
تلفن
+98 13 3336 9002
فکس
+98 13 3336 9070
ایمیل
17shahrivar@gums.ac.ir
آدرس صفحه وب

حمایت کنندگان / منابع مالی

1

حمایت کننده مالی
نام سازمان / نهاد
دانشگاه علوم پزشکی رشت
نام کامل فرد مسوول
شادمان نعمتی
آدرس خیابان
بیمارستان 17 شهریور. خیابان شهید سیادتی. خیابان نامجو
شهر
رشت
استان
گیلان
کد پستی
4144654839
تلفن
+98 13 3336 9002
فکس
ایمیل
research@gums.ac.ir
آدرس صفحه وب
ردیف بودجه
کد بودجه
آیا منبع مالی همان سازمان یا نهاد حمایت کننده مالی است؟
بلی
عنوان منبع مالی
دانشگاه علوم پزشکی رشت
درصد تامین مالی مطالعه توسط این منبع
100
بخش عمومی یا خصوصی
عمومی
مبدا اعتبار از داخل یا خارج کشور
داخلی
طبقه بندی منابع اعتبار خارجی
خالی
کشور مبدا
طبقه بندی موسسه تامین کننده اعتبار
دانشگاهی

فرد مسوول پاسخگویی عمومی کارآزمایی

اطلاعات تماس
نام سازمان / نهاد
دانشگاه علوم پزشکی رشت
نام کامل فرد مسوول
حمیدرضا بادلی
موقعیت شغلی
استادیار
آخرین مدرک تحصیلی
فوق تخصص
سایر حوزه‌های کاری/تخصص‌ها
کودکان
آدرس خیابان
بیمارستان 17 شهریور.خیابان شهید سیادتی.خیابان نامجو
شهر
رشت
استان
گیلان
کد پستی
4144654839
تلفن
+98 13 3336 9002
فکس
+98 13 3336 9070
ایمیل
badeli@gums.ac.ir

فرد مسوول پاسخگویی علمی مطالعه

اطلاعات تماس
نام سازمان / نهاد
دانشگاه علوم پزشکی رشت
نام کامل فرد مسوول
حمیدرضا بادلی
موقعیت شغلی
استادیار
آخرین مدرک تحصیلی
فوق تخصص
سایر حوزه‌های کاری/تخصص‌ها
کودکان
آدرس خیابان
بیمارستان 17 شهریور.خیابان شهید سیادتی.خیابان نامجو
شهر
رشت
استان
گیلان
کد پستی
4144654839
تلفن
+98 13 3336 9391
فکس
ایمیل
badeli@gums.ac.ir

فرد مسوول به‌روز رسانی اطلاعات

اطلاعات تماس
نام سازمان / نهاد
دانشگاه علوم پزشکی رشت
نام کامل فرد مسوول
حمیدرضا بادلی
موقعیت شغلی
دانشیار
آخرین مدرک تحصیلی
فوق تخصص
سایر حوزه‌های کاری/تخصص‌ها
کودکان
آدرس خیابان
بیمارستان 17 شهریور.خیابان شهید سیادتی.خیابان نامجو
شهر
رشت
استان
گیلان

کد پستی
414465839
تلفن
+98 13 3336 9002
ایمیل
badeli@gums.ac.ir

برنامه انتشار

فایل داده شرکت کنندگان (IPD)
خیر - برنامه‌ای برای انتشار آن وجود ندارد
توجیه/علت عدم تصمیم/عدم انتشار IPD
به دلیل مسایل اخلاقی و محرمانه بودن اطلاعات
پروتکل مطالعه
خیر - برنامه‌ای برای انتشار آن وجود ندارد
نقشه آنالیز آماری
خیر - برنامه‌ای برای انتشار آن وجود ندارد
فرم رضایتنامه آگاهانه
خیر - برنامه‌ای برای انتشار آن وجود ندارد
گزارش مطالعه بالینی
خیر - برنامه‌ای برای انتشار آن وجود ندارد
کدهای استفاده شده در آنالیز
خیر - برنامه‌ای برای انتشار آن وجود ندارد
نظام دسته‌بندی داده (دیکشنری داده)
خیر - برنامه‌ای برای انتشار آن وجود ندارد
