# Supplementary material for: Strengthening the success rate of suprapubic aspiration in infants by integrating point-of-care ultrasonography guidance: A parallel-randomized clinical trial
Source: PLoS One. 2021 Jul 15;16(7):e0254703. doi: 10.1371/journal.pone.0254703 (PMC8282064; doi:10.1371/journal.pone.0254703)
Supplement: S3 File — (DOCX) [file pone.0254703.s004.docx]

We confirm that the clinical trial protocol we have included as Supplementary Information is the version that was submitted to and approved by your ethics committee before the trial began

You can find the Persian and English version as below

**چکیده پروتکل**

**هدف ازمطالعه**

تعیین و مقایسه میزان موفقیت سه روش نمونه گیری ادرار با کاتتر

آسپیراسیون سوپراپوبیک با و بدون گایدنس در شیرخواران مشکوک به

عفونت ادراری

**طراحی**

کارآزمایی بالینی دارای گروه کنترل

**نحوه و محل انجام مطالعه**

نوزادان و شیرخواران بستری شده در بیمارستان 17 شهریور مشکوک

به عفونت ادراری 30 دقیقه پس از شیرخوردن بر اساس بلاک های

تصادفی به سه گروه درمانی A,B ,C تقسیم بندی میشوند. گروه : A

نمونه گیری با کاتترمثانه گروه B:نمونه گیری با آسپیراسیون

سوپراپوبیک گروه C:نمونه گیری سوپراپوبیک با گاید اولتراسونوگرافی

تعداد دفعات تلاش برای نمونه گیری حداکثر سه بار است

**شرکت کنندگان/شرایط ورود و عدم ورود**

شرایط ورود: کلیه نوزادان و شیرخواران کوچکتر از سه ماه مشکوک

به عفونت ادراری شرایط عدم ورود: عفونت پوست محل مورد بررسی

اختلالات آناتومیک دستگاه ادراری اختلالات خونریزی دهنده و

ترومبوسیتوپنی اصلاح نشده شکم و احشا شکمی برجسته

**گروههای مداخله**

گروه A : نمونه گیری با کاتترمثانه گروه B:نمونه گیری با آسپیراسیون

سوپراپوبیک گروه C:نمونه گیری سوپراپوبیک با گاید اولتراسونوگرافی

**متغیرهای پیامد اصلی**

میزان موفقیت کلی هر روش نمونه گیری ادرار

**کمیته اخلاق**

**نام کمیته اخلاق**

کمیته اخلاق دانشگاه علوم پزشکی گیلانٍ

**آدرس خیابان**

بیمارستان 17 شهریور.خیابان شهید سیادتی.خیابان نامجو

**شهر**

رشت

**استان**

گیلان

**کد پستی**

4144654839

**تاریخ تایید**

2016-12-15 , ۲۵ / ۰۹ / ۱۳۹۵

**کد کمیته اخلاق**

IR.GUMS.REC.1395.365

**Protocol summary**

**Study aim**

To determine and compare the success rate of three

urine sampling methods with catheterization, suprapubic

aspiration with and without guidance in infants

suspicious to urinary tract infection

**Design**

clinical trial with control group

**Settings and conduct**

Neonates and infants hospitalized in 17 Shahrivar

Hospital suspicious to Urinary Tract Infection are divided

into 3 therapeutic groups: A, B, and C according to block

randomization. Group A: Sampling with Catheterization

Group B: Sampling with Suprapubic Aspiration Group C:

Sampling with Ultrasonography-guided Suprapubic

Aspiration There is a maximum limit of three-time efforts

for sampling

**Participants/Inclusion and exclusion criteria**

Entry condition: All the Neonates and Infants younger

than 3 month suspicious to Urinary Tract Infection Noentry

conditions: Infection of the examed -skin site

Anatomical disorders of Urinary Tract Bleeding Disorders

and Uncorrected Thrombocytopenia Enlarged abdomen

and Inrta-abdominal Viscera

**Intervention groups**

Group A: Catheterization Group B: Suprapubic Aspiration

Group C: Ultrasonography-guided Suprapubic Aspiration

**Main outcome variables**

Overall success rate of each sampling method

**Ethics committee**

**Name of ethics committee**

Ethics committee of Guilan University of Medical

Sciences

**Street address**

Namjou Ave,Shadid Siadati St,Rasht

**City**

Rasht

**Province**

Guilan

**Postal code**

4144654839

**Approval date**

2016-12-15, 1395/09/25

**Ethics committee reference number**

IR.GUMS.REC.1395.365
